# Alopecia neoplastica: A manifestation of metastatic breast cancer

**DOI:** 10.1002/ski2.302

**Published:** 2023-10-13

**Authors:** Shohei Kitayama, Koji Katsuumi, Sumiko Takatsuka, Chizuko Kanbayashi, Tadamichi Shimizu, Tatsuya Takenouchi

**Affiliations:** ^1^ Division of Dermatology Niigata Cancer Center Hospital Niigata Japan; ^2^ Department of Dermatology Faculty of Medicine Academic Assembly University of Toyama Toyama Japan; ^3^ Department of Breast Oncology Niigata Cancer Center Hospital Niigata Japan

## Abstract

We herein report a typical case of alopecia neoplastica secondary to breast cancer. Alopecia neoplastica is a rare form of alopecia resulting from metastasis of a primary tumour to the scalp and is often misdiagnosed as alopecia areata.

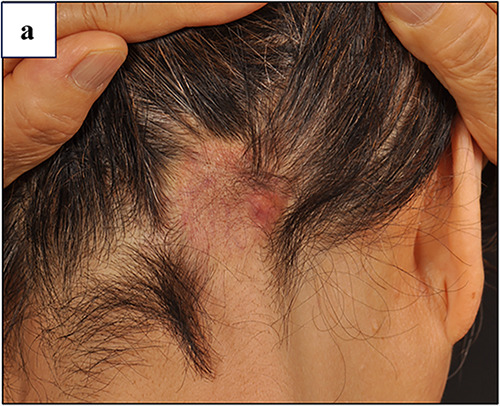

## Introduction

**FIGURE 1:**
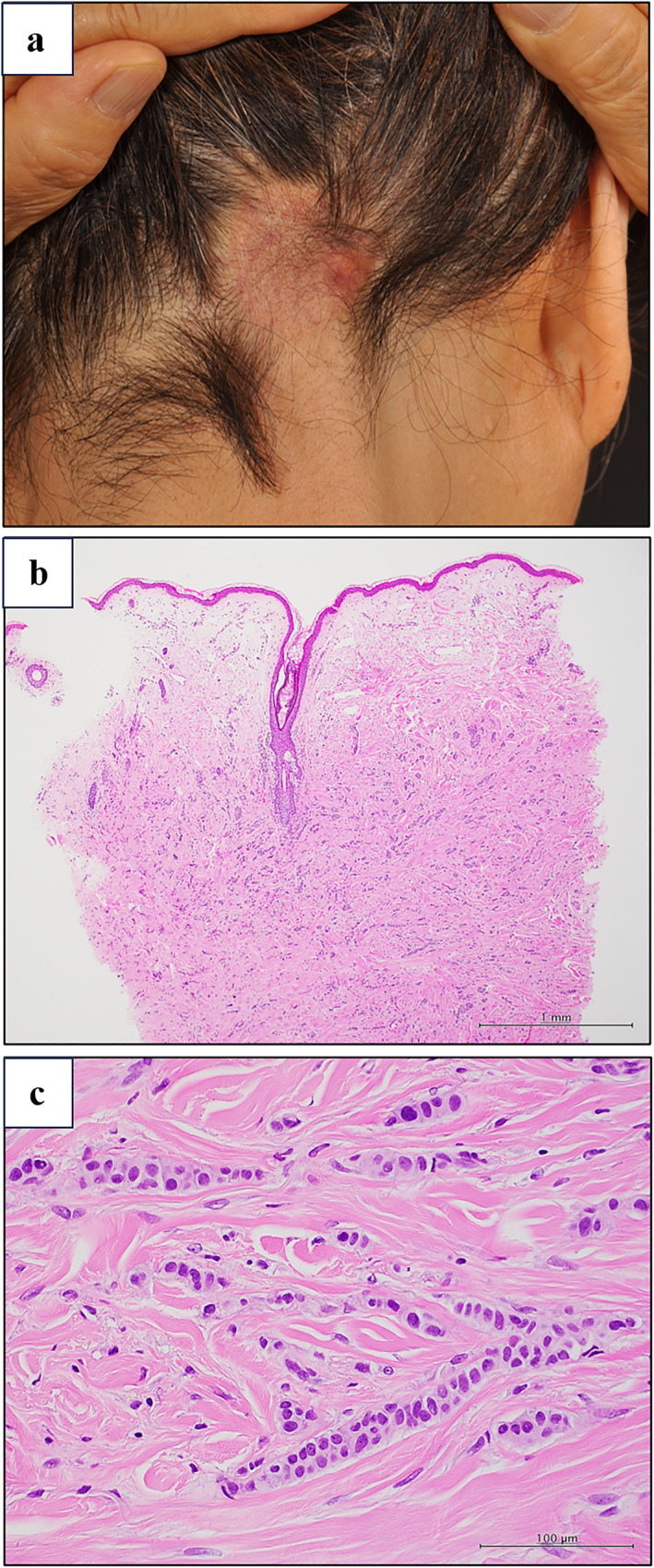
(a) The clinical findings of the patient at the initial presentation. A physical examination revealed a well‐defined, nonpruritic erythematous plaque with alopecia affected the occipital area. (b, c) A histopathological examination of an erythematous plaque revealed tumour cells distributed between dense collagen stroma in an Indian file pattern (haematoxylin‐eosin staining).

A 75‐year‐old woman presented with a 5‐year history of localised hair loss on the occipital scalp. The patient had a well‐defined erythematous plaque with alopecia (Figure 1a). Twelve years earlier, she underwent mastectomy for right breast invasive ductal carcinoma and was subsequently treated with anastrozole. A histopathological analysis confirmed breast cancer metastasis (Figure 1b,c). The patient was diagnosed with alopecia neoplastica. Anastrozole was continued, and the lesions remained unchanged during the 5‐year follow‐up period. Breast cancer is a leading cause of alopecia neoplastica.^1^ Careful dermatological examinations are required for alopecia in patients with a history of the disease.^1^, ^2^

## AUTHOR CONTRIBUTIONS

**Shohei Kitayama**: Conceptualization (lead); investigation (lead); writing – original draft (lead); writing – review & editing (equal). **Koji Katsuumi**: Investigation (supporting). **Sumiko Takatsuka**: Conceptualization (supporting); investigation (equal). **Chizuko Kanbayashi**: Investigation (lead). **Tadamichi Shimizu**: Supervision (lead). **Tatsuya Takenouchi**: Conceptualization (lead); supervision (lead); writing – review & editing (lead).

## CONFLICT OF INTEREST STATEMENT

None to declare.

## ETHICS STATEMENT

Not applicable.

## Data Availability

The data that support the findings of this study are available on request to the corresponding author. The data are not publicly available due to patient's privacy reasons.
